# The Impact of ABO Blood Type on Hemoglobin Decline After Total Hip Arthroplasty

**DOI:** 10.3390/jcm15020515

**Published:** 2026-01-08

**Authors:** Toshiyuki Kawai, Yaichiro Okuzu, Koji Goto, Yutaka Kuroda, Yugo Morita, Shuichi Matsuda

**Affiliations:** Department of Orthopaedic Surgery, Graduate School of Medicine, Kyoto University, 54 Kawahara-cho, Shogoin, Sakyo-ku, Kyoto City 606-8507, Japan; yokuzu@kuhp.kyoto-u.ac.jp (Y.O.);

**Keywords:** blood type, anemia, total hip arthroplasty, von Willebrand factor

## Abstract

**Background**: ABO blood type was reported to have a profound influence on hemostasis. This study aimed to determine the association between ABO blood type and the hemoglobin drop after total hip arthroplasty (THA). **Methods**: We retrospectively reviewed the changes in hemoglobin after elective primary THA. Demographic characteristics were analyzed for 794 eligible THAs. Changes in hemoglobin at postoperative days 1, 7, and 14 relative to the preoperative level were analyzed for 777 THAs that did not require allogenic blood transfusion (ABT). The effects of blood type were examined using a multivariate regression model and a propensity score matching model. **Results**: The hemoglobin drop was largest at 7 days, and the values differed significantly between type O cases and non-type O cases (2.68 ± 1.08 g/dL vs. 2.41 ± 1.02 g/dL; *p* = 0.0013). In the multivariate model, blood type O was identified as an independent factor for larger hemoglobin drop at 7 days (*p* = 0.015). Lower body mass index, non-hybrid THA, higher preoperative hemoglobin level, direct lateral approach, and prophylactic use of factor Xa inhibitor were also identified as independent risk factors for larger hemoglobin drop. After successful matching of 232 THAs in type O patients with 232 THAs in non-type O patients, hemoglobin drop at 7 days was significantly larger in type O patients (−2.44 ± 1.05 g/dL vs. −2.70 ± 1.05 g/dL, *p* = 0.0092). **Conclusions**: Blood type O was independently associated with a slightly greater postoperative hemoglobin decline after primary THA; however, the absolute between-group difference was small and was not accompanied by a higher allogenic transfusion rate. Therefore, ABO blood type may represent a minor risk marker and should be interpreted in the context of clinically more relevant bleeding- and hemodilution-related factors (e.g., perioperative anticoagulant/antiplatelet therapy and underlying coagulopathies).

## 1. Introduction

The ABO blood group system for red blood cell membrane antigens was established by Karl Landsteiner in the early 1900s [1]. The ABO blood group antigens are highly expressed on the surfaces of cells and tissues, including platelets, vascular endothelia, and epithelial surfaces [2], and are associated with various diseases and conditions. Recent studies have implicated ABO blood types as a potential risk factor for diseases such as cancer [3], myocardial infarction [4], acute kidney injury [5], and venous thromboembolism (VTE) [4]. ABO blood types were also reported to have a profound influence on hemostasis [3,4,5,6]. Previous studies indicated that non-type O patients, especially type AB patients, had twice the risk of VTE development compared with type O patients [4,6], which may imply that type O patients are prone to bleeding. Several studies have investigated the coagulation profiles for different blood types. Patients with blood types A, B, and AB had 25–30% higher plasma levels of von Willebrand factor (vWF) [7,8] and factor VIII (FVIII) [8] than patients with blood type O, indicating that type O patients could have an increased risk of bleeding.

Other studies demonstrated that individuals with blood type O had increased postpartum blood loss [9,10], higher risk of upper gastrointestinal hemorrhage [11], and greater likelihood of developing hemorrhage during extracorporeal membrane oxygenation therapy [12]. Blood type O was also associated with significantly elevated risks of overall mortality and exsanguination-related death in patients with severe trauma [13] and isolated severe abdominal trauma [14].

A previous report indicated an association of ABO blood type with postoperative bleeding volume in patients undergoing total hip arthroplasty (THA) [15]. In that short report, blood loss after THA was suggested to be larger in type O patients than in non-type O patients [15]. However, the study included only 30 type O patients (all women) and did not adjust for patient demographic characteristics and surgical factors in the analyses. Therefore, studies with larger cohorts that allow adjustment for confounding factors are necessary to clarify the effects of ABO blood type on blood loss after THA.

The present study aimed to determine the associations between ABO blood type and progression of anemia in patients undergoing THA. We hypothesized that type O patients would experience a larger hemoglobin (Hb) drop after THA than non-type O patients. To test this hypothesis, we assessed the associations between ABO blood type and Hb drop after THA using a multivariate regression model to adjust for the effects of confounding factors and a propensity score matching model.

## 2. Patients and Methods

**Figure 1:**
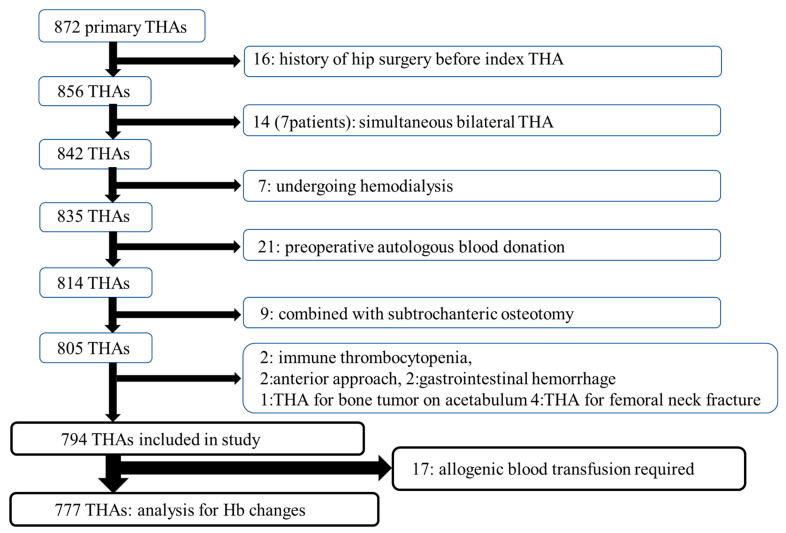
Flowchart showing the inclusion and exclusion of patients for the present study.

We retrospectively reviewed 872 consecutive primary THAs performed between May 2014 and December 2022 in our institutional database of THA cases. Of the 872 procedures, 95 were excluded because the patients had a history of hip surgery, had simultaneous bilateral procedures, were undergoing hemodialysis, donated autologous blood preoperatively, had subtrochanteric osteotomy during the index THA, had immune thrombocytopenia, had major gastrointestinal hemorrhage after THA, had an anterior approach, had a bone tumor on the acetabulum, or underwent THA for a femoral neck fracture (Figure 1). All patients provided written informed consent, and the study protocol was approved by the institutional review board of our hospital. This retrospective study was conducted with approval from the institutional review board on 19 May 2014. The database is maintained by five orthopedic surgeons, with coding accuracy reviewed at two-month intervals. All data were handled in accordance with applicable privacy regulations, and patient confidentiality was strictly maintained. Among the remaining 794 THAs, 17 (2.1%) received an allogenic blood transfusion (ABT) on the day of surgery or within 7 days after surgery. The remaining 777 THAs were available for the analysis of Hb changes after surgery.

As a prophylactic anticoagulant, the factor Xa inhibitor edoxaban could be administered for relatively inactive patients, but the use of the drug was dependent on the surgeon’s preference. The choice of dose (15 mg or 30 mg) was subject to recommendation from a pharmacist based on the patient’s body weight and renal function. The drug was started on postoperative day 2 and continued for 14 days.

The necessity for ABT was based on a standard protocol for patients with Hb < 7.0 g/dL or Hb < 8.0 g/dL with symptoms of vertigo, persistent fatigue, hypotension, or a history of cardiovascular disease. The decision for ABT was also based on patient consent. These transfusion thresholds were used as a clinically actionable definition of severe postoperative anemia requiring treatment in our standard practice. Because there is no universally accepted hemoglobin cutoff to define postoperative anemia after THA, we did not introduce an additional binary definition of “postoperative anemia”; instead, anemia progression was evaluated using postoperative hemoglobin decline from the preoperative baseline as a continuous outcome.

The Hb changes at postoperative days 1, 7, and 14 relative to the preoperative level were measured and expressed as D1-Pre, D7-Pre, and D14-Pre, respectively. Although blood tests were also carried between postoperative days 2 and 6, the data for that period were not analyzed because of the inconsistent timing among the patients. The preoperative blood tests were completed in the outpatient unit at 1 month before surgery. The intraoperative blood loss was measured based on the amount of blood in the gauze and suction bottles. Intraoperative blood loss was calculated as the sum of the net volume in the suction canister (after subtracting irrigation fluid, as appropriate) and the volume of blood absorbed by gauze/sponges, quantified using a gravimetric method (wet weight − dry weight; assuming 1 g ≈ 1 mL). Although this approach is commonly used to quantify visible intraoperative blood loss, the resulting value should be regarded as an estimate rather than an exact measurement. In particular, admixture of irrigation fluid and variability in sponge/gauze saturation may introduce measurement error.

### 2.1. Surgical Procedure

Five experienced hip surgeons performed the primary THAs through a mini-incision anterolateral approach or direct lateral approach (Hardinge) in the lateral decubitus position. The surgery was performed under general anesthesia with or without epidural anesthesia in most cases; however, spinal anesthesia was also used, especially during the COVID-19 pandemic. The type of THA was selected according to the surgeon’s preference and categorized as cemented, cementless, or hybrid (cementless acetabular component and cemented femoral component).

A closed suction drain was used in all cases until December 2017 and no cases after January 2019. From January 2018 to December 2018, a drainage tube was used according to the surgeon’s preference. To minimize perioperative blood loss, meticulous hemostasis was achieved using electrocautery throughout the procedure. Preoperative or intraoperative systemic tranexamic acid was not administered during the study period; however, all patients received 1 g of topical (intra-articular) tranexamic acid immediately after skin closure (via the drainage tube when used or via a 20-gauge catheter). A cell saver, representing an autologous transfusion derived from intraoperative blood loss using a cell salvage device, was administered to patients in the operation room immediately after surgery according to the surgeon’s preference.

### 2.2. Statistical Analysis

The homogeneity of variance among the groups at each time point was assessed by the Bartlett test. Differences in proportions were calculated using the Pearson chi-square test. For comparisons between two groups, Student’s *t*-test was used for variables with a normal distribution and the Mann–Whitney U test was used for variables with a non-normal distribution. For comparisons of more than two groups, one-way analysis of variance followed by a post hoc Tukey–Kramer multiple comparison test was applied when the variance was homogeneous and the Kruskal–Wallis test followed by a post hoc Steel–Dwass test was applied when the variance was not homogeneous.

Univariate and multivariate regression analyses were performed to determine the independent associations of D7-Pre Hb change with sex, age, body mass index (BMI), indication (primary osteoarthritis [OA]), type of THA (hybrid), preoperative Hb, operation time, surgical approach (mini-incision anterolateral), type of anesthesia (spinal + epidural), use of postoperative drainage tube, use of cell saver, use of anticoagulant before surgery, prophylactic use of factor Xa inhibitor after surgery for prevention of deep vein thrombosis, and blood type (type O or non-type O). Interactions were quantified using variance inflation factors, with values of 5–10 considered to indicate collinearity. D7-Pre Hb change was employed as the representative value because the anemia at postoperative day 7 was assumed to be the most severe among the three time points. The level of statistical significance was set at *p* < 0.05. Logistic regression was conducted to compute the propensity of blood type O. Patient demographic characteristics (age, sex, BMI, indication for THA, type of THA, preoperative Hb, operation time, surgical approach, use of postoperative drainage tube, use of cell saver, use of anticoagulant before surgery, and use of factor Xa inhibitor) were employed as covariates for the propensity score. Type O patients were matched non-type O patients using calipers with a width equal to 0.2× standard deviation of the logit of the propensity score. A 1:1 ratio was used for matching. Standardized mean differences (SMDs) for all covariates were estimated before and after matching, and balance was considered to be achieved for SMD < 0.1 [16]. Success of matching was judged by the proportion of patients matched and the balance of covariates after matching. The sample size required for between-group comparisons was calculated using bilateral alpha risk of 5%, 80% power, standard deviation of 1.00 g/dL, and difference of 0.3 g/dL for each blood type at 7 days after surgery by reference to our previous preliminary analysis on anemia after THA. At least 360 cases (180 type O and 180 non-type O) were required for matching. With an anticipated proportion of 38% for individuals with type O blood in Japan [17], we assumed that at least 480 cases were needed for the analyses. All statistical analyses were performed using JMP Pro 15 software (SAS Institute, Cary, NC, USA).

## 3. Results

A total of 794 THAs were included for the analysis of demographic characteristics in procedures with and without postoperative ABT (Table 1). The 777 procedures (97.9%) that did not require ABT were further analyzed for the effects of ABO blood type on postoperative Hb changes. The ABT rates did not differ significantly among the blood types (*p* = 0.83).

Table 2 shows the demographic data and Table 3 demonstrates postoperative Hb changes for each blood type. The largest Hb drop occurred at 7 days postoperatively, with values of 2.41 ± 1.02 g/dL for non-type O cases and 2.68 ± 1.08 g/dL for type O (*p* = 0.0013) (Table 3). The D1-Pre Hb and D7-Pre Hb changes differed significantly between non-type O cases and type O cases at every time point.

In the univariate models, younger age, higher preoperative Hb, smaller intraoperative blood loss, and diagnosis of developmental dysplasia of the hip or primary OA were associated with lower rate of ABT, while blood type was not associated with ABT (Table 1). Univariate regression analyses performed with D7-Pre Hb change as the dependent variable showed that blood type O, male sex, non-hybrid THA, larger preoperative Hb, direct lateral approach (versus mini-incision anterolateral), and prophylactic use of factor Xa inhibitor were associated with larger Hb drop (Table 4). The multivariate regression analysis identified blood type O as an independent factor associated with larger Hb drop after adjustment for confounding factors (Table 4). Smaller BMI, non-hybrid THA, higher preoperative Hb, direct lateral approach, and prophylactic use of factor Xa inhibitor were also identified as independent risk factors for larger Hb drop after THA.

In the comparisons between all 541 non-type O patients and 236 type O patients, hemoglobin decline was significantly greater in type O patients at postoperative day 1 (D1-Pre: −1.99 ± 0.97 vs. −1.76 ± 0.91 g/dL, *p* = 0.0017), day 7 (D7-Pre: −2.68 ± 1.08 vs. −2.41 ± 1.02 g/dL, *p* = 0.0013), and day 14 (D14-Pre: −2.39 ± 0.99 vs. −2.18 ± 1.00 g/dL, *p* = 0.0074) (Table 5). After propensity score matching (232 pairs), the D7–Pre hemoglobin decline remained significantly greater in type O patients than in matched non-type O patients (−2.70 ± 1.05 vs. −2.44 ± 1.05 g/dL, *p* = 0.0092) (Table 5).

## 4. Discussion

In this retrospective study on patients who underwent elective primary THA, the Hb drop after THA was larger in type O patients compared with non-type O patients. The Hb drop at postoperative day 7 was the largest among the time points examined. The multivariate regression and propensity score matching analyses confirmed that the D7-Pre Hb drop in type O patients was significantly larger than that in non-type O patients. To our knowledge, this is the first study to report an association between ABO blood type and Hb drop after THA after adjustment for confounding factors. A recent study emphasized that surgeons should risk-stratify their patients undergoing total joint arthroplasty with regard to anemia [18]. Although the difference in Hb drops between the matched non-O and matched O groups was small (2.41 g/dL vs. 2.68 g/dL), surgeons need to be aware of this risk factor because it is known that patients with anemia have a two- to three-fold increased risk of complications, including poor wound healing and impaired mobilization [19]. Importantly, the between-group difference in hemoglobin decline was small and was not accompanied by a higher allogenic transfusion rate under our institutional transfusion protocol (Hb < 7.0 g/dL, or Hb < 8.0 g/dL with symptoms). Therefore, the transfusion criteria serve as a clinically meaningful threshold for severe anemia requiring treatment, whereas hemoglobin decline was used in this study to quantify anemia progression as a continuous measure.

Several studies reported that type O patients had 25–30% lower plasma vWF levels than non-type O patients, which may increase their risk of hemorrhage [7,20] because vWF has essential roles for primary hemostasis by mediating adhesion of blood platelets to the subendothelium of damaged vessel walls and promoting aggregation of activated platelets. Furthermore, vWF acts as a carrier of FVIII involved in clotting activity and protects it against premature proteolysis [8,21,22]. However, several differences in the mechanisms of hemostasis according to blood type remain unknown. Further studies are needed to clarify the role of the blood types in maintaining hemostasis.

Although the multivariate analysis identified blood type O as an independent risk factor for postoperative Hb drop, the ABT rates did not differ significantly. However, the rates of ABT have the possibility of a beta error. Because the present study had a low rate of ABT after primary THA (2.1%), studies with larger cohorts are required to clarify the effects of blood type on the rate of ABT after THA. Detailed analyses of other risk factors for ABT were also difficult because of the low incidence of ABT (17 cases) in this cohort, which prevented the performance of logistic regression analyses to identify risk factors for ABT.

The multivariate model showed that prophylactic use of edoxaban (factor Xa inhibitor) was associated with larger Hb drop. This finding is consistent with previous studies showing that edoxaban administration after THA resulted in more cases with anemia [23,24].

While some previous studies found that blood loss and ABT rate were significantly increased when a closed suction drain was used in THA [25,26], a propensity score-matched cohort study showed that suction drainage was not associated with a difference in blood loss after THA [27]. In the present study, the Hb changes after THA did not differ between procedures with and without use of a closed suction drain.

Higher BMI was reported to be associated with larger blood loss [28], but lower risk of transfusion after THA [29]. In the present study, higher BMI was associated with smaller Hb drop. Combined with the previous findings, patients with higher BMI may lose more blood, but show a smaller Hb change because of their larger circulating blood volume.

Previous studies found a higher risk of bleeding in type O patients who experienced upper gastrointestinal hemorrhage [11]. Blood type O was also associated with higher postpartum blood loss compared with the other blood types. Specifically, Kahr et al. [30] found that women with blood type O had greater postpartum blood loss, Drukker et al. [9] found that women with blood type O had a greater risk of postpartum hemorrhage, and Bade et al. [10] found that women with blood type O had significantly more blood loss after caesarean delivery and higher risk for blood transfusion. In the present study, the Hb drop after THA was significantly larger in type O patients than in non-type O patients, consistent with the majority of previous studies.

Other reports indicated an increased risk of VTE in individuals with blood type AB after total joint arthroplasties [31], and during pregnancy and puerperium [32]. These differences could be explained by the finding that plasma vWF levels were lowest for blood type O, followed by type A, type B, and finally type AB [7].

There are several limitations to the present study. First, we only evaluated the phenotype in the ABO blood groups and not the genotypes. The highest levels of clotting factors FVIII and vWF were observed for the AA/AB/BB genotype, with intermediate levels for the AO/BO genotype, and low levels for the OO genotype [33,34]. Phenotype A in the present study would have included both the AO and AA genotypes. Therefore, the differences may have been larger if the comparisons had been performed among genotypes AA, BB, or AB and OO. Moreover, the effects of the Rh system were not analyzed because none of the patients in the cohort had Rh-negative blood. Although blood type was classified according to the conventional ABO system in this study, it should be noted that the underlying biological differences are more precisely explained by the ABH histo-blood group system, in which the presence or absence of A and B antigens represents differential modification of the H antigen. This framework may better account for the observed differences in postoperative hemoglobin decline, given the established association between ABH antigen expression and von Willebrand factor metabolism. Second, in the propensity score matching analyses, the D1-Pre and D14-Pre Hb drops tended to be larger in type O patients, but the differences were not significant (*p* = 0.0648 for D1-Pre and *p* = 0.0762 for D14-Pre). For the study, the required sample size was calculated to ensure sufficient numbers of cases to examine the difference at the time when the most severe anemia was anticipated. To strictly examine the differences at postoperative days 1 and 14, cohorts with larger numbers of patients are needed. Third, postoperative hemoglobin decline reflects not only surgical blood loss but also postoperative bleeding and perioperative hemodilution. In our database, quantitative postoperative drain output and detailed perioperative fluid balance were not available or not consistently recorded; therefore, these variables could not be incorporated into the multivariable regression or propensity score-matching models. Moreover, although we adjusted for preoperative anticoagulant use and postoperative factor Xa inhibitor prophylaxis, other bleeding-related comorbidities and medications (e.g., coagulation disorders, hepatic dysfunction, chronic kidney disease, hypertension, and antiplatelet therapy) were not fully captured. Accordingly, residual confounding may remain, and the magnitude of the observed association between ABO blood type and postoperative hemoglobin decline should be interpreted with caution. In addition, intraoperative blood loss (often reported as EBL), even when derived from suction canister volume and gauze/sponge weighing, is an imperfect surrogate for true blood loss and is subject to measurement error (e.g., irrigation fluid admixture and variability in sponge saturation). Therefore, the intraoperative blood loss values in this study should be interpreted with caution. Fourth, this study was retrospective in design and is therefore subject to inherent limitations, including potential selection bias, information bias, and residual confounding, as well as incomplete or inconsistently recorded variables in the institutional database. In addition, because we excluded patients with major coagulation abnormalities or extreme bleeding risk (e.g., immune thrombocytopenia and patients undergoing hemodialysis), the findings may not be generalizable to higher-risk populations, and the magnitude and clinical relevance of the association between ABO blood type and postoperative hemoglobin decline may differ in such settings. Finally, patients who had ABT were excluded from the analysis of the Hb drops after THA. However, in the study, the excluded patients with ABT were similarly distributed among the blood types, and therefore this process would not have greatly affected the results.

## 5. Conclusions

In this retrospective cohort, blood type O was independently associated with a modestly greater postoperative hemoglobin decline after primary THA; however, the magnitude of the difference was small and did not translate into a higher rate of allogenic blood transfusion. Accordingly, the clinical relevance of ABO blood type alone is likely limited, and perioperative management should continue to prioritize established, clinically important determinants of bleeding and postoperative anemia, such as preoperative anticoagulant/antiplatelet therapy and underlying coagulopathies, as well as postoperative bleeding and fluid-related hemodilution. Future studies incorporating quantitative postoperative bleeding measures and detailed perioperative fluid management are warranted to clarify the incremental value of ABO blood type for risk stratification.

## Figures and Tables

**Table 1:** Demographic characteristics of the patients with and without allogenic blood transfusion after surgery.

		Without Transfusion (n = 777)	Allogenic Blood Transfusion Used [17]	*p*-Value
Blood type	A	278 (97.5%)	7 (2.5%)	0.8315 ^a^
	B	186 (97.4%)	5 (2.6%)	
	AB	77 (98.7%)	1 (1.3%)	
	O	236 (97.9%)	4 (1.7%)	
Age (years)		63.9 ± 12.3 (17–89)	70.4 ± 13.8 (40–87)	0.0338 ^b^
BMI (kg/m^2^)		23.9 ± 4.2 (12.2–41.8)	22.1 ± 2.02 (17.5, 25.0)	0.0832 ^c^
Male sex (%)		158/777 (20.3%)	2/17 (11.8)	0.3835 ^a^
Indication for THA	DDH	508 (65.4%)	9 (52.9%)	0.0280 ^a^
	Primary OA	105 (13.5%)	0 (0%)	
	ONFH	130 (16.7%)	5(29.4%)	
	others	34 (4.4%)	3 (17.7%)	
Type of THA	Cement	27 (3.47%)	1 (5.9%)	0.7378 ^a^
	Cementless	187 (24.1%)	5 (29.4%)	
	Hybrid	563 (72.5%)	11 (64.7%)	
Preoperative Hb (g/dL)		13.07 ± 1.39 (8.0–19.0)	11.07 ± 1.30 (8.3–12.9)	<0.0001 ^b^
Operation time (minutes)		93.4 ± 27.1 (40–280)	102.9 ± 28.6 (60–157)	0.1556 ^b^
Approach (mini-anterolateral)		685/777 (88.2%)	15/17 (88.2%)	0.9924 ^a^
Postoperative drainage tube		423/777 (54.4%)	13/17 (76.5%)	0.0709 ^a^
Use of cell saver		527/777 (67.8%)	11/17 (64.7%)	0.7855 ^a^
On anticoagulant		67/777 (8.6%)	1/17 (5.88%)	0.6896 ^a^
Factor Xa inhibitor after surgery		267/777 (34.4)	7/17 (41.2%)	0.5588 ^a^
Intraoperative blood loss (mL)		167.5 ± 116.8 (5–900)	290.0 ± 148.0 (120–744)	<0.0001 ^b^

Data are shown as mean ± standard deviation (range) or ratio (%). BMI: body mass index, DDH: developmental dysplasia of the hip, OA: osteoarthritis, THA: total hip arthroplasty, ONFH: osteonecrosis of the femoral head. ^a^: Pearson chi-square test. ^b^: Student’s *t*-test. ^c^: Mann–Whitney U test.

**Table 2:** Patient demographic characteristics and hemoglobin changes after surgery for each blood type.

		All (n = 777)	AB (n = 77)	A (n = 278)	B (n = 186)	O (n = 236)	*p*-Value
Age (years)		63.9 ± 12.3 (17–89)	64.9 ± 11.0 (23–84)	63.6 ± 23.6 (17–89)	64.2 ± 12.4 (26–84)	63.4 ± 13.0 (17–86)	0.54 ^a^
BMI (kg/m^2^)		23.9 ± 4.2 (12.2–41.8)	23.7 ± 4.0 (16.3–35.1)	24.6 ± 4.6 (15.4–41.8)	23.5 ± 4.1 (13.2–39.7)	23.5 ± 3.8 (12.2–36.8)	0.028 ^b,c^
Male sex (%)		158/777 (%)	16/77 (%)	64/278 (%)	31/186 (%)	47/236 (%)	0.42 ^d^
Indication for THA	DDH	508 (65.4%)	51 (66.2%)	177 (63.7%)	125 (67.2%)	156 (66.1%)	0.8328 ^d^
	Primary OA	105 (13.5%)	9 (11.7%)	39 (14.0%)	26 (14.0%)	31 (13.1%)	
	ONFH	130 (16.7%)	13 (16.9%)	49 (17.6%)	32 (17.2%)	36 (15.3%)	
	others	34 (4.4%)	4 (5.2%)	13 (4.7%)	3 (1.6%)	13 (5.5%)	
Type of THA	Cement	27 (3.47%)	4 (5.2%)	9 (3.2%)	1 (0.5%)	13 (5.5%)	0.090 ^d^
	Cementless	187 (24.1%)	20 (26.0%)	62 (22.3%)	42 (22.6%)	63 (26.7%)	
	Hybrid	563 (72.5%)	53 (68.8%)	207 (74.5%)	143 (76.9%)	160 (67.8%)	
Preoperative Hb (g/dL)		13.1 ± 1.4 (8.0–19.0)	13.0± 1.5 (10.1–17.9)	12.9 ± 1.5 (8.0–17.7)	13.1 ± 1.3 (9.9–19.0)	13.2 ± 1.3 (9.7–16.8)	0.149 ^a^
Operation time (minutes)		93.4 ± 27.1 (40–280)	95.2 ± 32.0 (40–233)	94.5 ± 29.9 (42–280)	90.4 ± 23.2 (43–253)	94.0 ± 24.6 (47–219)	0.619 ^b^
Approach (mini-anterolateral)		685/777 (88.2%)	65/77 (84.4%)	243/278 (87.4%)	173/186 (93.0%)	204/236 (86.4%)	0.0870 ^d^
Postoperative drainage tube		423/777	45/77 (58.4%)	138/278 (49.7%)	97/186 (52.2%)	143/236 (60.6%)	0.069 ^d^
Use of cell saver		527/777 (67.8%)	55/77 (71.4%)	178/278 (64.0%)	128/186 (68.8%)	166/236 (70.3%)	0.382 ^d^
On anticoagulant		67/777 (8.6%)	11/77 (14.3%)	26/278 (9.4%)	13/186 (7.0%)	17/236 (7.2%)	0.207 ^d^
Factor Xa inhibitor after surgery		267/777 (34.4)	24/77 (31.2%)	93/278 (33.5%)	78/186 (41.9%)	72/236 (30.5%)	0.081 ^d^
Intraoperative blood loss (mL)		167.5 ± 116.8 (5–900)	167.5 ± 122.0 (5–760)	179.2 ± 127.7 (5–900)	144.7 ± 90.1 (5–488)	171.7 ± 118.4 (5–835)	0.041 ^b,e^

Data are shown as mean ± standard deviation (range) or ratio (%) BMI: body mass index, DDH: developmental dysplasia of the hip, OA: osteoarthritis, THA: total hip arthroplasty, ONFH: osteonecrosis of the femoral head. ^a^: Analysis of variance. ^b^: Kruskal–Wallis test. ^c^: *p* = 0.0391, type A vs. type B by a post hoc Steel–Dwass test. ^d^: Pearson chi-square test. ^e^: *p* = 0.0375, type A vs. type B by a post hoc Steel–Dwass test.

**Table 3:** Hemoglobin changes after surgery for each blood type.

	All (n = 777)	AB (n = 77)	A (n = 278)	B (n = 186)	O (n = 236)	***p***-Value
D1-Pre Hb drop (g/dL)	−1.83 ± 0.94 (−4.7, 1.8)	−1.77 ± 0.91 (−4.6, 0.5)	−1.68 ± 0.93 (−4.5, 1.8)	−1.87 ± 0.88 (−4.6, 0.5)	−1.98 ± 0.97 (−4.7, 0.0)	0.0022 ^a,b^
D7-Pre Hb drop (g/dL)	−2.50 ± 1.04 (−5.9, 0.7)	−2.36 ± 1.10 (−5.9, 0.0)	−2.40 ± 1.05 (−5.9, 0.7)	−2.46 ± 0.95 (−5.4, 0.0)	−2.68 ± 1.08 (−5.8, 0.4)	0.0094 ^a,c^
D14-Pre Hb drop (g/dL)	−2.25 ± 1.00 (−5.5, 1.4)	−2.18 ± 0.94 (−5.4, −0.5)	−2.19 ± 1.07 (−5.0, 1.1)	−2.18 ± 0.91 (−5.5, 0.3)	−2.39 ± 0.99 (−5.5, 1.4)	0.0665 ^a^
	Non O (n = 541)				O (n = 236)	
D1-Pre Hb drop (g/dL)	−1.78 ± 1.06 (−4.6, 1.8)				−1.98 ± 0.97 (−4.7, 0.0)	0.0101 ^a^
D7-Pre Hb drop (g/dL)	−2.41 ± 1.02 (−5.9, 0.7)				−2.68 ± 1.08 (−5.8, 0.4)	0.0013 ^a^
D14-Pre Hb drop (g/dL)	−2.18 ± 1.00 (−5.5, 1.1)				−2.39 ± 0.99 (−5.5, 1.4)	0.0316 ^a^

^a^: Analysis of variance. ^b^: *p* = 0.0012, type A vs. type O by a post hoc Tukey–Kramer test. ^c^: *p* = 0.0119, type A vs. type O by a post hoc Tukey–Kramer test.

**Table 4:** Univariate and multivariate regression analyses performed with D7-Pre hemoglobin change as the dependent variable (n = 777).

	Univariate				Multivariate				
	*t*	Standard Error	Standardized Coefficients Beta	*p*-Value	*t*	Standard Error	Standardized Coefficients Beta	*p*-Value	VIF
**Blood type O**	−3.30	0.0811	−0.1178	0.0010	−2.45	0.0700	−0.0756	0.0146	1.0296
age	1.09	0.0031	0.0391	0.2761	−1.86	0.0028	−0.0625	0.0627	1.2150
Male sex	−3.68	0.0931	−0.1312	0.0002	1.70	0.0888	0.0579	0.0894	1.2523
BMI	0.22	0.0089	0.0078	0.8289	3.13	0.0080	0.1015	0.0018	1.1356
Primary OA	0.43	0.1097	0.0155	0.6663	0.34	0.0978	0.0108	0.7351	1.1079
Hybrid THA	3.67	0.0834	0.1309	0.0003	2.63	0.0800	0.0904	0.0086	1.2726
Preoperative Hb	−14.71	0.0239	−0.4677	<0.0001	−14.52	0.0254	−0.4918	<0.0001	1.2390
Operation time	−4.45	0.0014	−0.1581	<0.0001	−1.84	0.0014	−0.0675	0.0656	1.4455
Approach (mini-anterolateral)	6.44	0.1131	0.2256	<0.0001	4.66	0.1147	0.1662	<0.0001	1.3733
Anesthesia (spinal + epidural)	1.27	0.1858	0.0455	0.2056	1.45	0.1600	0.0451	0.1466	1.0405
Postoperative drainage tube	−1.40	0.0753	−0.0503	0.1612	0.69	0.0691	0.0229	0.4880	1.1794
Cell saver	0.28	0.0803	0.0099	0.7811	−0.50	0.0697	−0.0157	0.6153	1.0586
On anticoagulant	1.61	0.1334	0.0579	0.1072	−0.47	0.1189	−0.0150	0.6383	1.0982
Prophylactic Xa inhibitor	−2.70	0.0786	−0.0964	0.0072	−2.31	0.0711	−0.0749	0.0212	1.1369

BMI: body mass index, OA: osteoarthritis, THA: total hip arthroplasty, VIF: variance inflation factor.

**Table 5:** Demographic characteristics for non-type O patients and type O patients before and after propensity score matching.

		Non-O (n = 541)	O (n = 236)	*p*-Value	SMD	Matched Non-O (n = 232)	Matched O (n = 232)	*p*-Value	SMD
Age (years)		64.1 ± 12.0 (17–89)	63.4 ± 13.0 (17–86)	0.8358 ^a^	0.0541	63.5 ± 12.8 (23–89)	63.6 ± 12.8 (17–86)	0.8874 ^a^	0.0132
BMI (kg/m^2^)		24.1 ± 4.4 (13.2–41.8)	23.5 ± 3.8 (12.2–36.8)	0.2441 ^b^	0.1345	23.5 ± 4.4 (15.4, 41.8)	23.6 ± 3.7 (13.6, 36.8)	0.8895 ^a^	0.0129
Male sex		111/541 (20.5%)	47/236 (19.9%)	0.8479 ^c^	0.0049	40/232 (17.2%)	45/232 (19.4%)	0.5485 ^c^	0.0569
Indication for THA	DDH	352 (65.1%)	156 (66.1%)	0.5000 ^c^	0.0211	145 (62.5%)	155 (66.8%)	0.7031 ^c^	0.0900
	Primary OA	74 (13.7%)	31 (13.1%)			30 (12.9%)	30 (12.9%)		
	ONFH	94 (17.4%)	36 (15.3%)			42 (18.1%)	36 (15.5%)		
	others	21 (3.9%)	13 (5.5%)			15 (6.5%)	11 (4.7%)		
Type of THA	Cement	14/541 (2.59%)	13 (5.5%)	0.0576 ^c^	0.1482	11 (4.7%)	10 (4.3%)	0.9355 ^c^	0.0195
	Cementless	124/541 (22.9%)	63 (26.7%)			60 (25.9%)	63 (27.2%)		
	Hybrid	403/541 (74.5%)	160 (67.8%)			161 (69.4%)	159 (68.5%)		
Preoperative Hb (g/dL)		13.0 ± 1.4 (8.0–19.0)	13.2 ± 1.3 (9.7–16.8)	0.0514 ^a^	0.1530	13.2 ± 1.4 (8.0, 19.0)	13.2 ± 1.3 (9.7, 16.8)	0.9512 ^a^	0.0057
Operation time (minutes)		93.2 ± 28.1 (40–280)	94.0 ± 24.6 (47–219)	0.3156 ^b^	0.0329	93.1 ± 28.4 (43, 253)	93.8 ± 24.4 (47, 219)	0.7833 ^a^	0.0256
Approach (mini-anterolateral)		481/541 (88.9%)	204/236 (86.4%)	0.3273 ^c^	0.0760	206/232 (88.8%)	203/232 (87.5%)	0.6666 ^c^	0.0399
Postoperative drainage tube		280/541 (51.8%)	143/236 (60.6%)	0.0229 ^c^	0.1781	138/232 (59.5%)	141/232 (60.8%)	0.7761 ^c^	0.0255
Use of cell saver		361/541 (66.7%)	166/236 (70.3%)	0.3218 ^c^	0.0776	169/232 (72.8%)	164/232 (70.7%)	0.6061 ^c^	0.0477
On anticoagulant		50/541 (9.24%)	17/236 (7.2%)	0.3518 ^c^	0.0743	14/232 (6.0%)	16/232 (6.9%)	0.7058 ^c^	0.0353
Prophylactic Xa inhibitor after surgery		195/541 (36.0%)	72/236 (30.5%)	0.1351 ^c^	0.1169	70/232 (30.2%)	71/232 (30.6%)	0.9196 ^c^	0.0093
Intraoperative blood loss (mL)		165.7 ± 116.2 (5–900)	171.7± 118.4(5–835)	0.3528 ^a^		166.1 ± 109.3 (5, 692)	172.7 ± 118.6 (5, 835)	0.5325 ^a^	
D1-Pre Hb drop (g/dL)		−1.76 ± 0.91 (−4.6, 1.8)	−1.99 ± 0.97 (−4.7, 0)	0.0017 ^a^		−1.83 ± 0.89 (−4.6, 0.8)	−1.99 ± 0.96 (−4.7,0.0)	0.0648 ^a^	
D7-Pre Hb drop (g/dL)		−2.41 ± 1.02 (−5.0, 0.7)	−2.68 ± 1.08 (−5.8, 0.4)	0.0013 ^a^		−2.44 ± 1.05 (−5.4 0.7)	−2.70 ± 1.05 (−5.8, 0.4)	0.0092 ^a^	
D14-Pre Hb drop (g/dL)		−2.18 ± 1.00 (−5.5, 1.1)	−2.39 ± 0.99 (5.5, 1.4)	0.0074 ^a^		−2.24 ± 1.03 (−5.5, 1.1)	−2.41 ± 0.98 (−5.5, 1.4)	0.0762 ^a^	

Data are shown as mean ± standard deviation (range) or ratio (%). BMI: body mass index, DDH: developmental dysplasia of the hip, OA: osteoarthritis, THA: total hip arthroplasty, ONFH: osteonecrosis of the femoral head, SMD: standardized mean difference. ^a^: Student’s *t*-test. ^b^: Mann–Whitney U test. ^c^: Pearson chi-square test.

## Data Availability

The original contributions presented in this study are included in the article. Further inquiries can be directed to the corresponding author.

## Abbreviations

The following abbreviations are used in this manuscript:

THA	total hip arthroplasty
ABT	allogenic blood transfusion
VTE	venous thromboembolism
vWF	von Willebrand factor
FVIII	factor VIII
Hb	hemoglobin
BMI	body mass index
OA	osteoarthritis
